# A novel *TAFAZZIN* gene variant c.525_533del causing Barth syndrome and leading to heart transplantation: a case report

**DOI:** 10.3389/fped.2025.1634258

**Published:** 2025-08-18

**Authors:** Michał Krawiec, Joanna Śliwka, Szymon Pawlak, Michał Kapałka, Paulina Hamerling, Weronika Grzywacz, Wiktoria Hawel, Gabriela Skórska, Dorota Piekutowska-Abramczuk, Tomasz Hrapkowicz

**Affiliations:** ^1^ Faculty of Medical Sciences in Zabrze, Medical University of Silesia in Katowice, Zabrze, Poland; ^2^ Department of Cardiac, Vascular and Endovascular Surgery and Transplantology, Faculty of Medical Sciences in Zabrze, Medical University of Silesia in Katowice, Silesian Center for Heart Diseases in Zabrze, Zabrze, Poland; ^3^ Faculty of Medical Sciences in Katowice, Medical University of Silesia in Katowice, Katowice, Poland; ^4^ Department of Medical Genetics, The Children’s Memorial Health Institute, Warsaw, Poland

**Keywords:** Barth syndrome, *TAFAZZIN* gene, tafazzin, cardiolipin, cardiomyopathy, heart transplant

## Abstract

**Introduction:**

Barth syndrome (BTHS) is an ultra-rare genetic disease caused by a mutation in the *TAFAZZIN* gene, located on the X chromosome. This gene codes for the protein tafazzin, which is involved in the metabolism of the mitochondrial phospholipid - cardiolipin. Symptoms of this genetic defect include dilated cardiomyopathy (DCM), skeletal myopathy, neutropenia, growth retardation, reduced cholesterol levels, increased serum lactic acid levels, and hypoglycemia in the neonatal period.

**Case description:**

A Caucasian boy with DCM and left ventricular non-compaction associated with BTHS, caused by a previously unreported variant in the *TAFAZZIN* gene: NM_000116.4:c.525_533del; NP_000107.1:p.(His176_Phe178del) at NC_000023.11:g.154419607_154419615del, in the exon 6. Due to the patient's heart failure, a mechanical circulatory support (MCS) system was required, followed by orthotopic heart transplantation (OHT). Because of the presence of neutropenia, standard immunosuppressive therapy had to be modified in the postoperative period.

**Conclusions:**

A previously unreported mutation is presented, leading to BTHS. This disease can have severe cardiovascular manifestations, requiring MCS and OHT.

## Introduction

Barth syndrome (BTHS; MIM 302060) is an ultra-rare genetic disorder with an incidence of 1 in 400,000 to 1 in 1,000,000 births (1). Approximately 250 cases of the disease have been described worldwide since 1983 (1). The cause of BTHS is a mutation in the *TAFAZZIN* gene, located on the X chromosome (1, 2). This gene encodes the protein tafazzin, and its mutation results in impaired mitochondrial metabolism of the phospholipid cardiolipin. Tafazzin is a non-specific phospholipid-lysophospholipid transacylase responsible for modifying the structure of cardiolipin, a key component of the mitochondrial membrane. This leads to severe abnormalities, like a disruption of the electron transport chain, increased mitochondrial reactive oxygen species (both in the mechanism of enhanced production and accumulation), dysregulation of CoA-dependent metabolism, and dysfunction of the citric acid cycle (1–4).

Symptoms of this genetic disease include dilated cardiomyopathy (DCM), skeletal myopathy, neutropenia, growth retardation, reduced cholesterol level, increased serum lactic acid levels, and hypoglycemia in the neonatal period (1–5). This case study presents a male patient with DCM and left ventricular non-compaction (LVNC) attributed to BTHS, caused by a previously unreported variant of the c.525_533del p.(His176_Phe178del) in the *TAFAZZIN* gene.

## Case description

A Caucasian boy was delivered by cesarean section at 36 weeks' gestation due to fetal growth restriction and placental insufficiency. Patient's birth weight was 2,450 g and he was 50 cm in length. The patient was from his mother's third pregnancy and second delivery. The mother had previously one miscarriage, and the boy's older brother had died at 5 months of age due to DCM with myocardial non-compaction.

Prenatal testing had shown thickened right ventricular myocardium. On the second day of life, the patient was admitted to the cardiology unit due to bradycardia (heart rate dropping to about 76 beats per minute). At that time, decreased muscle tone and a heart rate of 90–100 bpm with drops to 80 bpm were observed. Blood tests revealed leukopenia (7.59 × 10^3^/µl; normal range: 9.40–34.00 × 10^3^/µl) with a low number of eosinophils (0.04 × 10^3^/µl; normal range: 0.2–0.4 × 10^3^/µl) and an elevated number of monocytes (1.55 × 10^3^/µl; normal range: 0.10–1.10 × 10^3^/µl). Echocardiography revealed right ventricular myocardial thickening and decreased contractility of both ventricles, with a reduced left ventricular ejection fraction (LVEF) of approximately 55%. The left ventricular diameter measured 1.79 cm in diastole (LVDd) and 1.34 cm in systole (LVDs). A patent foramen ovale (PFO) and increased LV trabeculation was also identified. Based on these findings, DCM was diagnosed, and LVNC was suspected.

After treatment with captopril, spironolactone, and carvedilol, normalization of heart rate and an increase in LVEF to about 80% were achieved.

Based on the clinical symptoms, BTHS was suspected, and a urine test for organic acids using gas chromatography coupled to mass spectrometry (GC-MS) was ordered. The test showed elevated concentrations of lactic acid, 2-ketoglutaric acid, p-hydroxyphenyllactic acid, and p-hydroxyphenylpyruvic acid; no succinylacetone was found.

In the first months of the patient's life, delayed motor and speech development were observed. At the age of 14 months, he was hospitalized for sepsis of staphylococcal etiology and agranulocytosis.

During the diagnostic process, genomic DNA was isolated from blood leukocytes using standard procedures, including phenol/chloroform extraction and automated DNA extraction (MagNA Pure LC 2.0, Roche). Mutation analysis was performed using Sanger sequencing of PCR-amplified exons 2–11 of the *TAFAZZIN* gene, including exon/intron boundaries, on a 3130 Genetic Analyzer (Applied Biosystems/Life Technologies, Foster City, CA).

During the diagnostic process, DNA sequencing was performed using the Sanger method with analysis of exons 2–11 of the *TAFAZZIN* gene. Numbering of revealed nucleotide changes was based on the reference sequence for the *TAFAZZIN* gene (hg38; NM_000116.5; NP_000107.1); position +1 corresponded to the A of the ATG translation initiation codon. The study revealed a deletion of (NM_000116.4:c.525_533del; NP_000107.1:p.(His176_Phe178del); NC_000023.11:g.154419607_154419615del) in exon 6 of the *TAFAZZIN* gene. This molecular variant c.525_533del.p(His176_Phe178del) had not been previously reported in The Human Gene Mutation Database (HGMD). The same mutation was found in the patient's mother, who was heterozygous (a carrier), and was subsequently inherited by the child.

**Figure 1:**
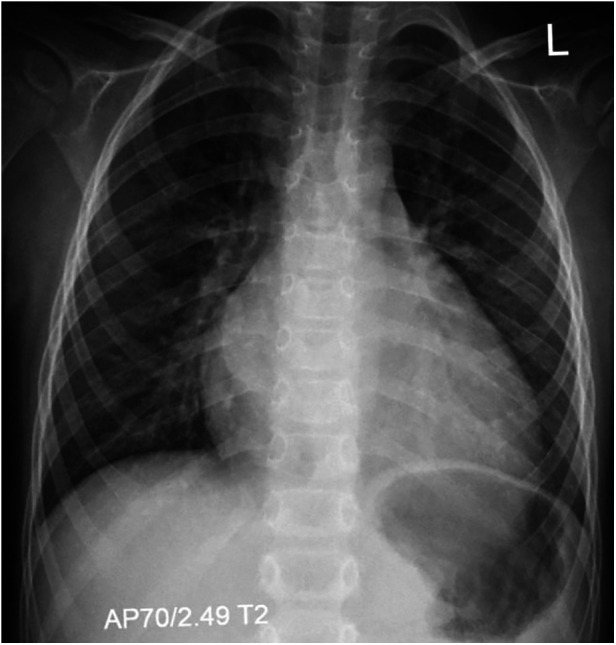
Chest x-ray (AP view) showing marked cardiomegaly consistent with dilated cardiomyopathy, without signs of pulmonary congestion.

**Figure 2:**
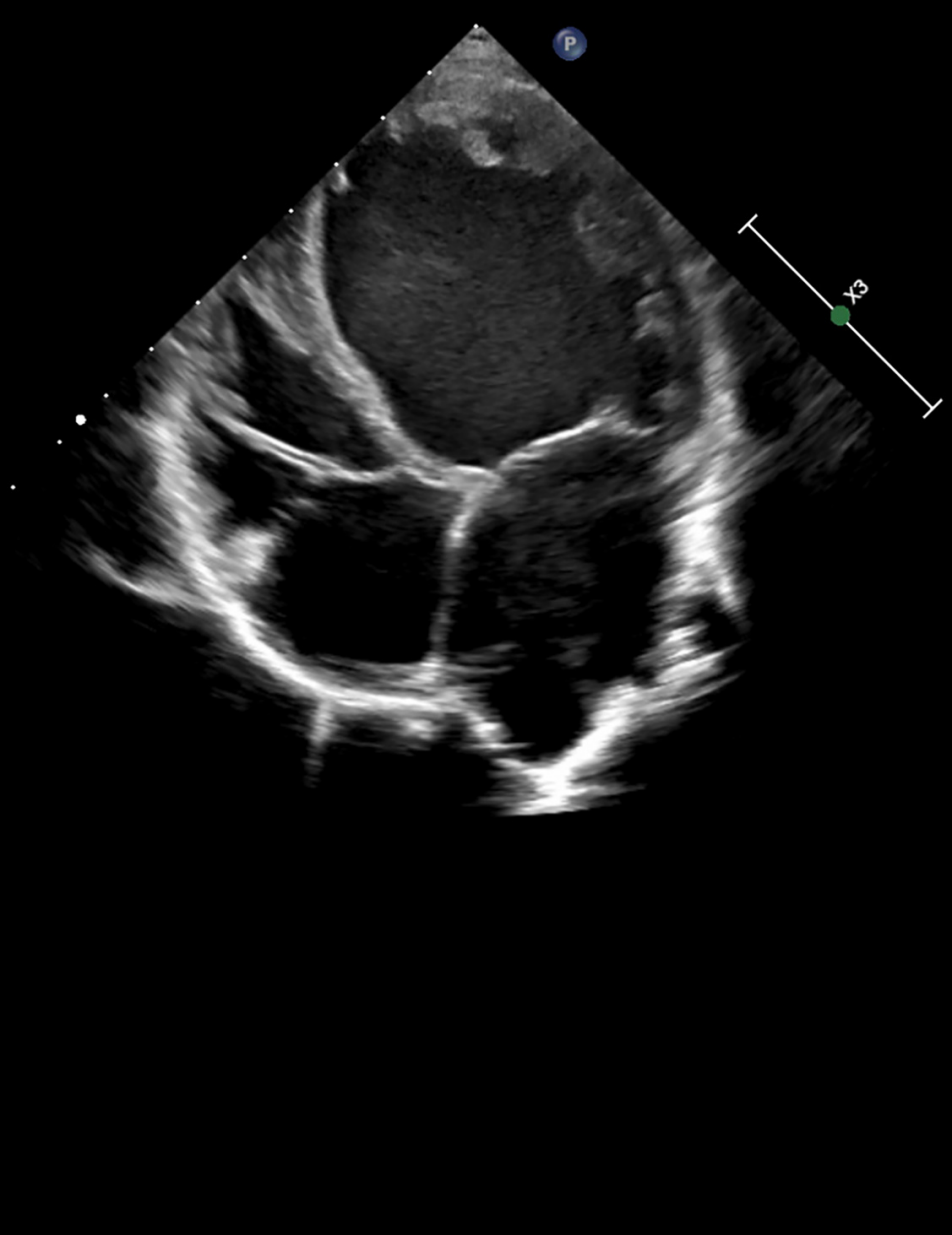
Echocardiographic image in a parasternal long-axis view showing the left ventricle, left atrium, and aortic root. Cardiac structures appear with clear wall delineation, suggesting preserved image quality for structural assessment.

At the age of 7, the boy was admitted to a cardiac center due to a sudden deterioration in his condition. On admission, the patient's weight was 17.4 kg and he was 115 cm tall (both values were <3 percentile). There was tachycardia (approximately 160 bpm), profuse vomiting, and a quiet systolic murmur on the left side of the sternum. Blood tests showed elevated NT-proBNP (10,064 pg/ml; normal <83 pg/ml) and troponin T (86 pg/ml; normal <14 pg/ml). Chest X-ray revealed an increased cardiac silhouette (Figure 1). An echocardiogram revealed a dilated left ventricle (LVDd: 4.73 cm – *Z*-score: 2.7; LVDs: 4.33 cm – *Z*-score: 5.22) with features of non-compaction, PFO, and small regurgitations on the mitral and tricuspid valves (Figure 2). The LVEF was about 18%. The electrocardiograms (ECG) showed non-specific intraventricular conduction abnormalities, ST-T segment inversion in the V5, V6 leads and a borderline corrected QT interval (QTc) of up to 453 ms (normal: <440 ms). The Holter ECG showed ten ventricular extrasystoles. Due to the patient's deteriorating condition, a left ventricular assist device (LVAD) was implanted as a bridge to orthotopic heart transplant (OHT).

During hospitalization, fluctuating levels of neutrophils (from 0.07 × 10^3^/µl to 8.34 × 10^3^/µl, often remaining below the lower limit of normal) and monocytes (from 0.60 × 10^3^/µl to 2.74 × 10^3^/µl, consistently above the upper limit of normal) were observed. Two times the LVAD chamber had to be exchanged due to fibrin deposits. Antibiotic therapy was required on several occasions: vancomycin due to *Clostridioides difficile* infection, ceftriaxone due to urinary tract infection and cloxacillin due to LVAD cannulation site infection. After 383 days of mechanical circulatory support, the patient underwent OHT.

After OHT, immunosuppression was implemented: methyloprednisone (in dose 50 mg twice daily), basiliximab (in dose 10 mg). On the day following the surgery, a neutropenia (2.19 × 10^3^/µl) was observed, leading to a temporary decision to withhold mycophenolate mofetil. Two days later, normal neutrophil levels were achieved. The tacrolimus in dose 2 mg was ordered. The mycophenolate mofetil was introduced on the fifth post-operative day at a dose of 250 mg twice daily. Additionally, on the fifth day after surgery, methyloprednisolone was substituted with prednisone in dose 20 mg. The patient was discharged home in good general condition after three weeks. Twenty months after surgery, the patient is well and has not required further cardiac interventions. The function of the transplanted heart was normal with an LVEF of 55%. During follow-up, no significant haematological disorders or infections were observed.

## Discussion

Cardiomyopathies are a heterogeneous group of myocardial disorders classified according to etiology, phenotypic features, and impact on cardiac function. In the category of primary genetic cardiomyopathies, hypertrophic cardiomyopathy (HCM), arrhythmogenic right ventricular cardiomyopathy (ARVC), and LVNC are distinguished. Cardiomyopathies of mixed etiology include DCM and restrictive cardiomyopathy (RCM), while peripartum and stress-induced cardiomyopathy (Tako-Tsubo syndrome) are classified as acquired cardiomyopathies. Secondary cardiomyopathies result from systemic diseases (6).

DCM, the most common cardiomyopathy in the general population (1:250–1:2500 people) with an incidence of 5–7 cases per 100,000 persons per year. DCM most often manifests itself between 20 and 60 years of age, but it is also the most common form of cardiomyopathy in children (more than 60% of childhood cardiomyopathies) (7). DCM is characterized by dilatation of the heart chambers with preserved wall thickness and systolic dysfunction, often leading to heart failure with reduced ejection fraction (8). The most common primary cardiomyopathy is HCM (9). Among primary cardiomyopathies, rare forms such as LVNC, which has an embryonic origin manifesting as significant trabeculation and development of intertrabecular spaces in the left ventricle, are also identified. The prevalence of LVNC in the general population is difficult to determine but is estimated to affect less than 1% of the population (6).

DCM is the most common form of cardiomyopathy leading to orthotopic heart transplantation (OHT) and represents a major cardiac complication in BTHS (2, 3). In the present case, prenatal thickening of the right ventricular myocardium and postnatal deterioration of biventricular contractility, with a reduced ejection fraction, indicate early cardiac involvement in the disease process. These findings are consistent with previous reports describing BTHS manifestations as early as the fetal period (10). LVNC is also a common phenotype in patients with BTHS, affecting 20%–50% of these patients (1, 4). Unlike other etiologies of DCM, where ventricular dilatation is usually progressive and associated with continuous impairment of systolic function, in BTHS it can be fluctuating, which may be related to the underlying mitochondrial dysfunction characteristic of the syndrome. In BTHS, despite the presence of DCM, a preserved or slightly reduced ejection fraction is often observed, distinguishing it from other causes of DCM (2, 5). In BTHS, left ventricular dilatation is not directly related to left ventricular weakness but may result from impaired relaxation and filling of the ventricle. This condition may be partially reversible, as evidenced by cases of improvement after supportive treatment (1, 4).

In the presented case, initial management centered on the administration of captopril, spironolactone, and carvedilol. This standard pharmacotherapy, in accordance with current clinical guidelines, led to the normalization of heart rhythm and a significant improvement in LVEF, reaching approximately 80%. This outcome aligns with existing evidence suggesting that patients with BTHS may respond favorably to conventional heart failure therapies, despite the absence of specific clinical trials evaluating the efficacy of these treatments in this population (1, 11, 12).

However, due to an acute clinical deterioration characterized by left ventricular dilation and a marked reduction in LVEF to approximately 18%, more advanced therapeutic interventions became necessary. LVAD implantation is typically employed as a bridging therapy to OHT (13), which is considered the definitive treatment option when other therapeutic modalities have proven insufficient (1). Available data indicate that approximately 14% of patients with BTHS ultimately require OHT (1).

The molecular etiology of BTHS is attributed to mutations in the *TAFAZZIN* gene, which encodes the tafazzin protein. This mutation disrupts the regulation of cardiolipin biosynthesis and remodelling within the inner mitochondrial membrane (1–5). Given the high mitochondrial density in organs such as the heart and skeletal muscle, mitochondrial dysfunction typically manifests as cardiomyopathy or skeletal myopathy (1).

**Figure 3:**
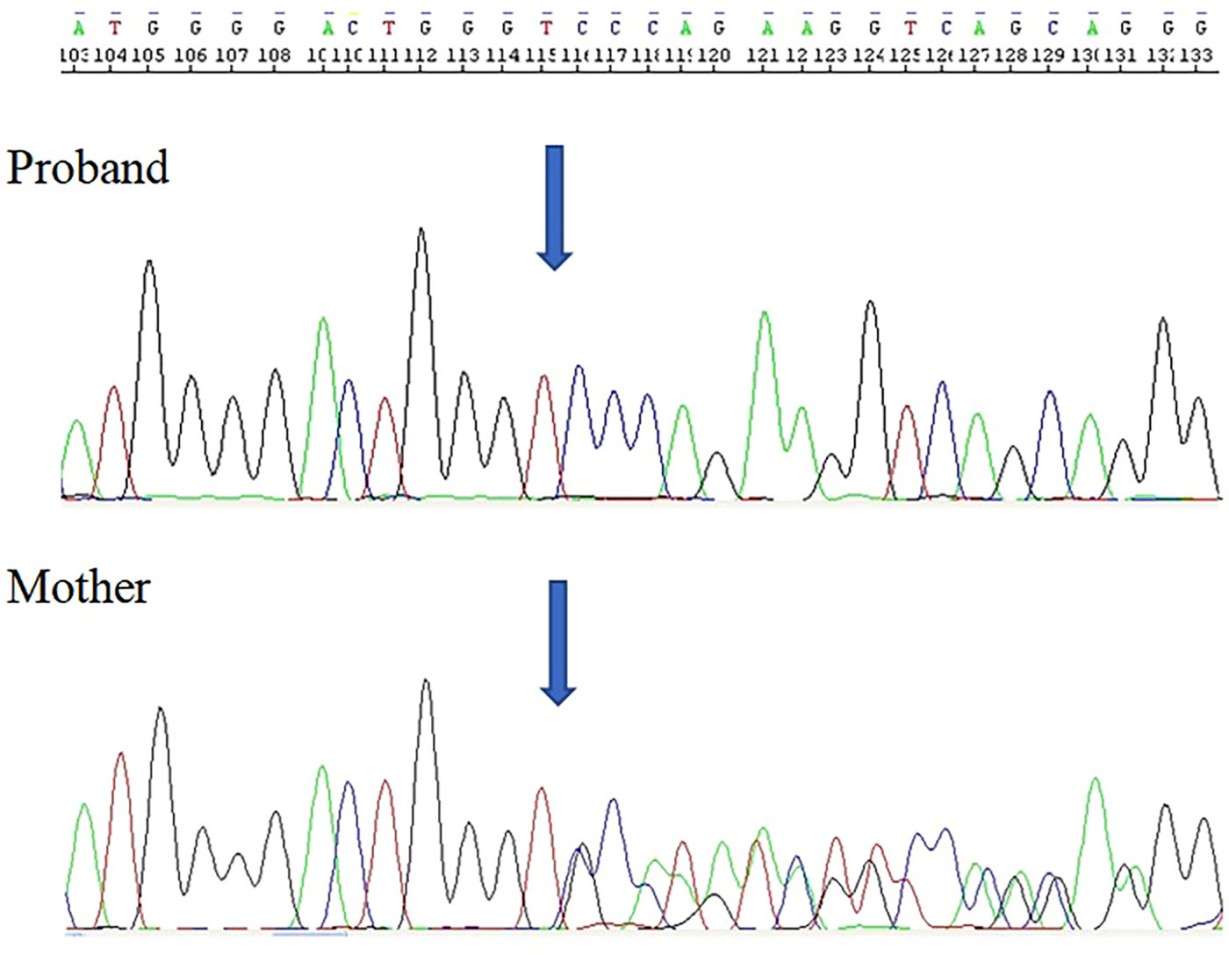
Electropherogram of the *TAFAZZIN* gene sequencing in the hemizygous patient revealed the in-frame deletion c.525_533del.p(His176_Phe178del).

The described proband, along with his mother, was found to harbor a previously unidentified gene mutation, c.525_533del.p(His176_Phe178del), located in exon 6 of the *TAFAZZIN* gene (Figure 3). This variant has not been previously reported in the Human Gene Mutation Database (HGMD) (14), the ClinVar database maintained by the National Center for Biotechnology Information (15), the Human *Tafazzin* Gene Variants Database curated by the Barth Syndrome Foundation (16) (accessed on 14th April 2025) and also in GnomAD v.4.1.0 database (17).

Variant is located in a region where there are missense (likely) pathogenic changes reported (chrX: 154419614T>A and chrX: 154419614T>C), both affects protein function (acyltransferase domain). MutationTaster algorithm predicts its deleterious effect (18).

**Figure 4:**
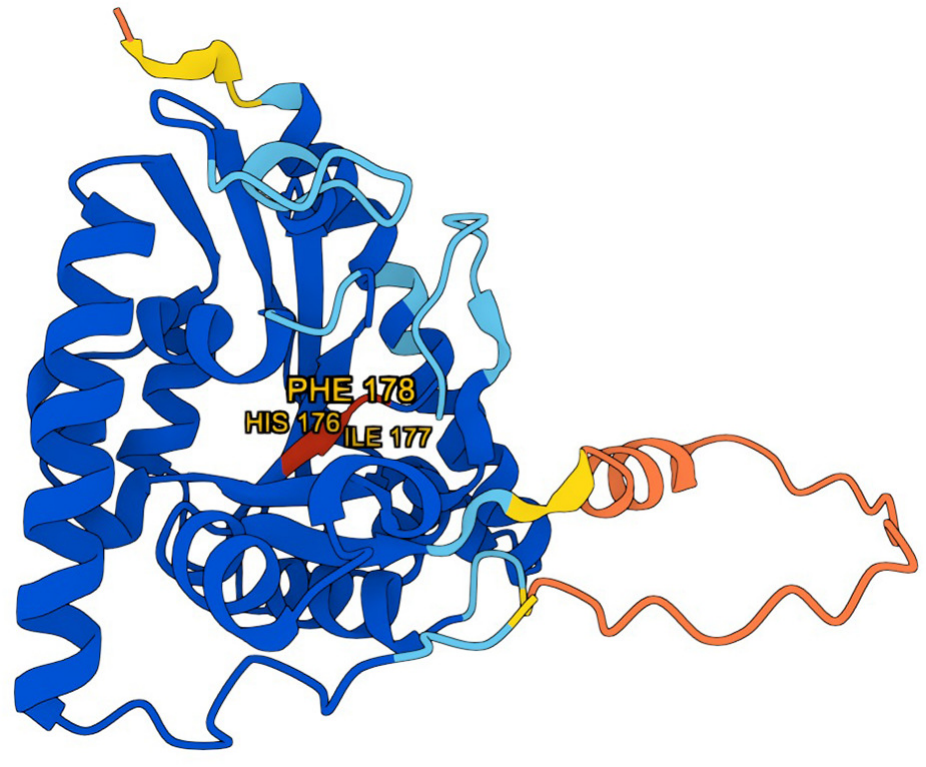
3D model of the tafazzin protein generated using AlphaFold (DeepMind), with amino acids affected by mutation marked in red. Model visualization is based on data provided by AlphaFold, [used under the Creative Commons Attribution 4.0 (CC BY 4.0)].

According to AlphaFold, the average missense pathogenicity scores were 0.995 for histidine, 0.876 for isoleucine, and 0.99 for phenylalanine, with a threshold of 0.564 above which pathogenicity is considered likely (19) (Figure 4).

The potential impact of the c.525_533del variant on splicing was assessed using SpliceAI and Pangolin. SpliceAI predicted a low-confidence donor site loss (score = 0.23 at position −2 bp relative to the variant), while Pangolin indicated minimal splice site loss (score = 0.05) and negligible splice site gain (score = 0.01). These findings suggest that the variant is unlikely to significantly disrupt canonical splicing or create cryptic splice sites (20).

According to Ensembl - all *TAFAZZIN* gene variants encoding the tafazzin protein contain the locus in which the mutation described in our study has occurred (21).

In silico conservation analysis using PhyloP100 revealed a score of 8.555 for the deleted region, indicating strong evolutionary conservation (22). This supports the pathogenic potential of the in-frame deletion.

According to American College of Medical Genetics and Genomics (ACMG) scoring this variant was classified as likely pathogenic: PM1 (located in a critical and well-established functional domain - active site of an enzyme); PM2 (absent from controls in Exome Sequencing Project, 1000 Genomes Project, or Exome Aggregation Consortium); PM4 (protein length changes as a result of in-frame deletions in a nonrepeat region) and PP3 (can be used only once in any evaluation of a variant) (23).

Analysis of the c.525_533del.p(His176_Phe178del) mutation revealed its impact at the amino acid level, altering a conserved region within the tafazzin protein. This protein functions as a phospholipid–lysophospholipid transacylase, essential for cardiolipin remodeling. Disruption of tafazzin activity leads to an accumulation of monolysocardiolipin and a decrease in mature cardiolipin levels, resulting in increased mitochondrial reactive oxygen species (ROS) production. Elevated ROS levels activate Ca^2+^/calmodulin-dependent protein kinase II (CaMKII), which phosphorylates ryanodine receptor 2 (RyR2), enhancing Ca^2+^ leakage from the sarcoplasmic reticulum. This cascade elevates diastolic Ca^2+^ levels and depletes sarcoplasmic reticulum Ca^2+^ stores, impairing cardiomyocyte contractility, relaxation, and development, thereby contributing to the pathophysiology of BTHS and DCM (1, 24).

This finding is particularly significant given the previously reported novel mutation c.83T > A, p.Val28Glu, in a Polish family (25). The challenges encountered in diagnosing BTHS, as highlighted by the previously reported familial mosaicism necessitating multi-tissue genetic testing, further emphasize the critical need for increased genetic diagnostic efforts. The rarity of diagnosed cases, coupled with the recurrent discovery of novel pathogenic variants in the Polish population, strongly advocates for a proactive approach to genetic screening.

Additional mutations within this region have been documented, underscoring the significance of this genomic segment in the pathogenesis of BTHS. A case involving a c.526C>T mutation (p.His176Tyr), described by Hirono et al., concerned a neonate diagnosed on the second day of life. The patient presented with heart failure, marked by an LVEF of 17%, ventricular tachycardia, tachypnoea, neutropenia, motor retardation, and 3-methylglutaconic aciduria, with no relevant family history. The patient succumbed to the condition at 5 months of age (26). Similarly, Thompson et al. reported a 4-year-old patient with the same mutation, presenting with delayed growth (*Z*-scores for height and weight of −3.25 and −2.60, respectively), increased LVDd and LVSd (*Z*-scores of 1.54 and 2.60, respectively), reduced left ventricular fractional shortening (29.06%), and elevated cardiolipin ratio (17.3; normal <0.2) along with raised methylglutaconic acid levels (1,048.5 nmol/L; normal range 162 ± 68 nmol/L) (27). Wang C. et al. described a case of fetal death due to congestive heart failure, attributed to a double pathogenic mutation involving both p.His176Tyr in the *TAFAZZIN* gene and p.Arg99His in the *KCNE3* gene, the latter being associated with Brugada syndrome (28).

Wang J. et al. reported the c.527A>G mutation (p.His176Arg) in twin brothers. These male infants were born with moderately low birth weights and presented with pneumonia and heart failure at 2.5 months of age, followed by a diagnosis of cardiomyopathy. Both exhibited hypotonia, motor delay, and growth retardation. Echocardiographic findings revealed reduced LVEF (45.6% and 36.2%), decreased left ventricular shortening fractions (22.1% and 16.7%), and LVDd Z-scores of 5.7 and 3.8, respectively. Additionally, a high, though sub-pathological, noncompaction/compaction (NC/C) ratio of 1.58 and 2.20 was observed. ECG revealed ST-T segment abnormalities and borderline QTc intervals (441 ms and 431 ms), alongside 3-methylglutaconic aciduria. Both patients died at 7 and 7.5 months of age (29).

Wang H. et al. reported a mutation at c.528A>C (p.His176Pro) (30).

The c.528_541 + 7del (r.spl) mutation is listed in the ClinVar database (31).

The c.532T>A mutation (p.Phe178Ile) was identified in a patient presenting with DCM, neutropenia, and growth retardation. The patient's brother was also affected, and the patient died at 9 months of age (32).

The c.532T>C mutation (p.Phe178Leu) is noted in the Human *Tafazzin* Gene Variants Database, based on an individual report (15).

The available data suggest that patients carrying mutations affecting at least one nucleotide within the region of the c.525_533del deletion, as observed in our patient, exhibit a similar constellation of symptoms: heart failure with reduced LVEF, DCM, tachypnea, prolonged QTc interval, ventricular extrasystoles, ST-T segment changes, growth retardation, motor delays, or neutropenia. Data regarding cardiolipin levels and 3-methylglutaconic aciduria in the described patient were not available. According to the literature, our patient with the c.525_533del deletion is the longest-lived patient (currently 10 years) and the only patient with a mutation in this region to have undergone OHT.

Neutropenia is a well-documented, dose-dependent adverse effect of mycophenolate mofetil (33). There are also reports describing abnormalities in both the number and morphology of neutrophils in heart transplant recipients treated with mycophenolate mofetil (34). It has been suggested that these morphological changes may result from inhibition of the enzyme inosine monophosphate dehydrogenase, leading to reduced *de novo* synthesis of guanosine nucleotides (35). Azathioprine is another immunosuppressive agent associated with a high risk of neutropenia (36); however, it was not administered in the case described. In patients with an increased risk of neutropenia, such as those with BTHS, immunosuppressive therapy should be managed with particular caution.

## Conclusions

The deletion c.525_533del.p(His176_Phe178del) is strongly suspected to be responsible for the clinical manifestations of BTHS. OHT remains the gold standard of treatment for advanced cardiomyopathy in this syndrome. Therefore, our findings, coupled with the limited number of diagnosed cases and the prevalence of novel *TAFAZZIN* variants in the Polish/Caucasian population, strongly underscore the critical need for increased awareness and the routine implementation of genetic diagnostics in individuals presenting with pathognomonic symptoms or a family history of such cardiac manifestations.

## Data Availability

The original contributions presented in the study are included in the article and the Supplementary Material. Further inquiries can be directed to the corresponding author.
